# CheXLocNet: Automatic localization of pneumothorax in chest radiographs using deep convolutional neural networks

**DOI:** 10.1371/journal.pone.0242013

**Published:** 2020-11-09

**Authors:** Hongyu Wang, Hong Gu, Pan Qin, Jia Wang

**Affiliations:** 1 Faculty of Electronic Information and Electrical Engineering, Dalian University of Technology, Dalian, Liaoning, China; 2 Department of Surgery, Second Hospital of Dalian Medical University, Dalian, Liaoning, China; Lingnan University, HONG KONG

## Abstract

**Background:**

Pneumothorax can lead to a life-threatening emergency. The experienced radiologists can offer precise diagnosis according to the chest radiographs. The localization of the pneumothorax lesions will help to quickly diagnose, which will be benefit for the patients in the underdevelopment areas lack of the experienced radiologists. In recent years, with the development of large neural network architectures and medical imaging datasets, deep learning methods have become a methodology of choice for analyzing medical images. The objective of this study was to the construct convolutional neural networks to localize the pneumothorax lesions in chest radiographs.

**Methods and findings:**

We developed a convolutional neural network, called CheXLocNet, for the segmentation of pneumothorax lesions. The SIIM-ACR Pneumothorax Segmentation dataset was used to train and validate CheXLocNets. The training dataset contained 2079 radiographs with the annotated lesion areas. We trained six CheXLocNets with various hyperparameters. Another 300 annotated radiographs were used to select parameters of these CheXLocNets as the validation set. We determined the optimal parameters by the AP_50_ (average precision at the intersection over union (IoU) equal to 0.50), a segmentation evaluation metric used by several well-known competitions. Then CheXLocNets were evaluated by a test set (1082 normal radiographs and 290 disease radiographs), based on the classification metrics: area under the receiver operating characteristic curve (AUC), sensitivity, specificity, and positive predictive value (PPV); segmentation metrics: IoU and Dice score. For the classification, CheXLocNet with best sensitivity produced an AUC of 0.87, sensitivity of 0.78 (95% CI 0.73-0.83), and specificity of 0.78 (95% CI 0.76-0.81). CheXLocNet with best specificity produced an AUC of 0.79, sensitivity of 0.46 (95% CI 0.40-0.52), and specificity of 0.92 (95% CI 0.90-0.94). For the segmentation, CheXLocNet with best sensitivity produced an IoU of 0.69 and Dice score of 0.72. CheXLocNet with best specificity produced an IoU of 0.77 and Dice score of 0.79. We combined them to form an ensemble CheXLocNet. The ensemble CheXLocNet produced an IoU of 0.81 and Dice score of 0.82. Our CheXLocNet succeeded in automatically detecting pneumothorax lesions, without any human guidance.

**Conclusions:**

In this study, we proposed a deep learning network, called, CheXLocNet, for the automatic segmentation of chest radiographs to detect pneumothorax. Our CheXLocNets generated accurate classification results and high-quality segmentation masks for the pneumothorax at the same time. This technology has the potential to improve healthcare delivery and increase access to chest radiograph expertise for the detection of diseases. Furthermore, the segmentation results can offer comprehensive geometric information of lesions, which can benefit monitoring the sequential development of lesions with high accuracy. Thus, CheXLocNets can be further extended to be a reliable clinical decision support tool. Although we used transfer learning in training CheXLocNet, the parameters of CheXLocNet was still large for the radiograph dataset. Further work is necessary to prune CheXLocNet suitable for the radiograph dataset.

## Introduction

Chest radiography is the most common and effective means for screening and diagnosing thoracic disease. It has been known that the radiologist and clinicians can be trained to make effective judgments after observing hundreds of chest radiographs [1]. However, it cannot be expected that the experienced radiologist and clinicians are available whenever and wherever possible, especially in the underdeveloped areas. Thus, a computer aid diagnosis system able to effectively detect the areas of pneumothorax in the radiographs can provide substantial benefits in the clinical diagnosis.

To days, deep learning algorithms have been widely applied to medical image analysis. Ciresan et al. used deep max-pooling convolutional neural networks (CNNs) to detect mitosis in breast histology images [2]. Ronneberger et al. developed U-net and won the ISBI cell tracking challenge 2015 [3]. Drozdzal et al. studied the influence of skip connections on fully convolutional network (FCN) for biomedical image segmentation [4]. Lopez-Garnier et al. designed a CNN for interpreting the digital images of Microscopic Observed Drug Susceptibility cultures [5]. Zhou et al. combined U-Nets of varying depths as UNet++ to improve the medical imaging segmentation performance of the fixed-depth U-Net [6]. Mzoughi et al. designed a 3D CNN layer with small kernels to merge both the local and global contextual information [7]. Shabanian et al. combined 2D U-nets into a 3D breast segmentation model with a suitable projection-fusing approach [8]. The deep learning models also have been trained for the classification tasks of chest radiographs. The deep learning models were developed for the classification of tuberculosis with an area under the receiver operating characteristic curve (AUC) of 0.99 [9]. Rajpurkar et al. developed a system that can classify 14 different diseases in chest radiographs [10]. They evaluated the algorithm against 9 practicing radiologists on a validation set and found it was comparable to practicing radiologists. Zech et al. evaluated the universality of CNN in the detection of pneumonia in radiographs from different hospital systems [11]. Their study reflected that CNNs robustly identified hospital system and department within a hospital. Taylor et al. trained CNN classifiers for pneumothorax in radiographs capable of detecting pneumothorax on a chest radiograph [12]. Salehinejad et al. used a deep convolutional generative adversarial network (DCGAN) to overcome the imbalanced radiographs dataset [13]. Nowadays, deep learning algorithms are widely used to analyze chest radiographs for COVID-19 diagnosis [14–16]. Zhu et al. employed CNNs to stage the lung disease severity of COVID-19 infection on portable chest radiographs [14]. Oh et al. proposed a patch-based CNN, to deal with a small COVID-19 radiograph dataset [15]. The lung areas were first extracted and then divided into patches for the COVID-19 classification network. Apostolopoulos et al. used transfer learning to overcome the insufficient amounts of COVID-19 dataset [16]. Comparing with classification, the localization of lesions can offer more information for the diagnose. Several deep learning methods have been developed for the classification as well as the localization task for diseases in chest radiographs. These localization methods were developed with weakly-supervised approaches using the weights and the feature maps of the classification networks [10, 15, 17]. The weakly-supervised methods cannot achieve the same accuracy as the fully-supervised ones [18]. Thus, the deep learning method for the precise localization of lesions in chest radiographs should be further investigated.

There are two popular methods frequently used in medical segmentation: Mask R-CNN and U-net [3, 6, 8, 19]. Mask R-CNN was developed from R-CNN [20–22]. R-CNN uses selective search to generate category-independent region proposals and then uses a deep CNN to classify the object proposals [20]. Faster R-CNN uses the region proposal network (RPN) to generate region proposals instead of selective search as well as extracting features [21]. A bounding-box regressor is added to Faster R-CNN parallel to the classifier based on R-CNN [21]. Mask R-CNN extends Faster R-CNN by adding a branch for segmentation in parallel with with the regressor branch [22]. U-Net is a U-shaped convolutional network that consists of an encoder and a decoder [3]. U-Net obtains rich segmentation features from the encoder module. The decoder module is to get a pixel level classification from the features learned by the encoder while connected with the encoder at different resolutions to recover missing information by the downsampling. Compared with U-net, Mask R-CNN seems to detect lesions better but could not segment as accurately [19].

In this work, we developed the deep network, called CheXLocNet, based on Mask R-CNN [22] for localizing lesions in chest radiographs with fully-supervision. CheXLocNet first localized a rough lesion area in a chest radiograph by using RPN. Then, it realized a more precise segmentation based on the rough localized area. The SIIM-ACR Pneumothorax Segmentation dataset was used to illustrate the effectiveness of our method.

## Materials and methods

### Data

We used the dataset for the SIIM-ACR Pneumothorax Segmentation Competition on Kaggle (https://www.kaggle.com/c/siim-acr-pneumothorax-segmentation/), which contains 12047 chest radiographs with pixel-level annotations. The dataset was separated into a 75%/12.5%/12.5% training/validation/test set split. The training set used to optimize the model parameters was of 2,079 positive and 7,250 negative radiographs. The validation set used to determine the optimal model from the candidates was of 300 positive and 1,046 negative radiographs. The test set used to evaluate the optimal model was of 290 positive and 1082 negative radiographs. Note that the test set was as same as the one used in the competition, which has no intersection with the training and validation set.

### Structure of CheXLocNets

Image segmentation is a kind of machine learning issue to predict the label for each pixel of images [22]. The segmentation method can be used for the localization of lesions in radiographs. CNNs have been widely applied to image segmentation [23]. We developed our CheXLocNet based on Mask R-CNN [22], which is a relatively concise and fast CNN for image segmentation. CheXLocNet mainly consisted of a backbone network, a RPN, a classification network and a mask network.

**Fig 1:**
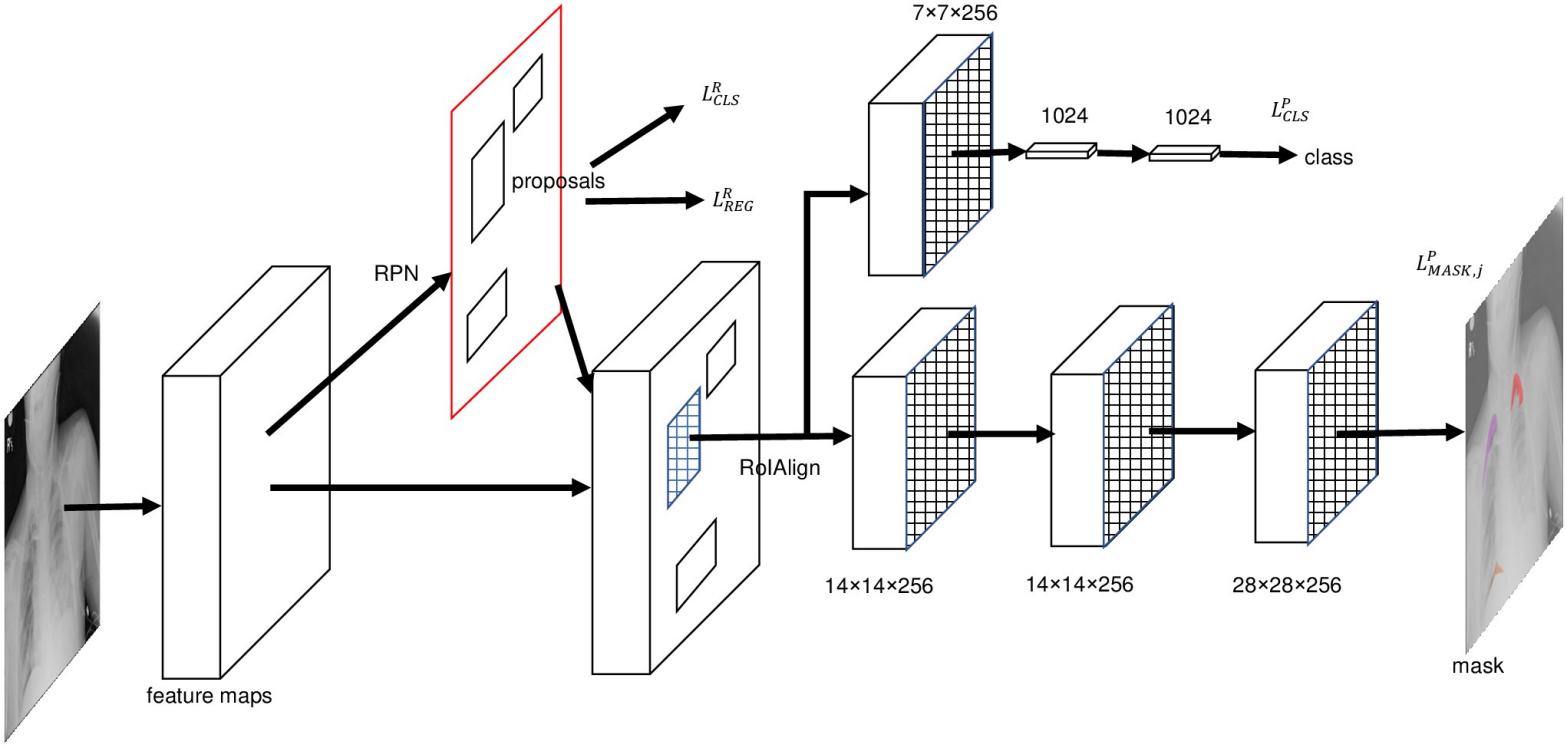
The framework of CheXLocNet. The features were extracted from the origin radiographs by the backbone network. RoI was screened out by RPN. RPN produced two losses $L_{CLS}^{R}$ and $L_{REG}^{R}$ during the training time. The classification network and the mask network produced their losses and predictions, respectively. The softmax was used for outputting the probability for being a lesion area for each RoI. The per-pixel sigmoid was used for outputting a mask. RoI, rectangular region of interest; RPN, region proposal network.

The backbone network was used for feature extraction over an entire image. We used the ResNet-50 with a feature pyramid network (FPN) as the backbone network [22, 24, 25]. ResNet-50 solves the degradation problem by adding identity connections to the convolution network [25]. FPN was used to take a top-down architecture with lateral connections to build high-level semantic feature maps at all scales [24]. RPN took an image without restrictions of size as input and output a set of rectangular regions of interest (RoIs) with FCN [26]. A 3 × 3 spatial convolution kernel slid over feature maps to generate input for RPN. At each sliding window location, RPN predicted multiple region proposals. The *k* proposals were relative to *k* reference boxes called anchors. An anchor was centered at the sliding window and was associated with a scale and aspect ratio. Before the classification network and the mask network, the RoIAlign [22] was used to convert RoI into a fixed size. The framework of CheXLocNet is shown in Fig 1. The classification network and the mask network were parallel networks to produce classification possibility and segmentation possibility. The decoder module is adopted to restore the high-level semantic features extracted from the feature encoder module.

### Loss function of CheXLocNets

RPN had two sibling output layers. The first sibling layer output objective probabilities of anchors. We defined ***t***_***i***_ = (*t*_*i*,*x*_, *t*_*i*,*y*_, *t*_*i*,*w*_, *t*_*i*,*h*_)^T^ as a vector denoting 4 parameterized coordinates of the ground-truth box associated with a positive anchor [20, 21]. And the bounding-box regression offsets output by the second sibling layer were defined as ${\hat{t}}_{i}=({\hat{t}}_{i,x},{\hat{t}}_{i,y},{\hat{t}}_{i,w},{\hat{t}}_{i,h}{)}^{T}$. The elements of ***t***_***i***_ and ${\hat{t}}_{i}$ were obtained as follow:

$$\begin{array}{cc} & {\hat{t}}_{i,x}=({\hat{x}}_{i}-x_{i,a})/w_{i,a},\;{\hat{t}}_{i,y}=({\hat{y}}_{i}-y_{i,a})/h_{i,a}, \end{array}$$ (1)

$$\begin{array}{cc} & {\hat{t}}_{i,w}={\hat{w}}_{i}/w_{i,a},\;{\hat{t}}_{i,h}={\hat{h}}_{i}/h_{i,a}, \end{array}$$ (2)

$$\begin{array}{cc} & t_{i,x}=(x_{i}-x_{i,a})/w_{i,a},\;t_{i,y}=(y_{i}-y_{i,a})/h_{i,a}, \end{array}$$ (3)

$$\begin{array}{cc} & t_{i,w}=w_{i}/w_{i,a},\;t_{i,h}=h_{i}/h_{i,a}, \end{array}$$ (4)

where *x*, *y*, *w*, and *h* denoted the box’s center coordinates and its width and height. Variables ${\hat{x}}_{i}$, *x*_*i*,*a*_, and *x*_*i*_ were for the predicted box, anchor box, and ground-truth box respectively (likewise for *y*, *w*, *h*). For the classification, we used the cross-entropy loss. For the regression, we used the Huber loss, a robust *L*_1_ loss which was less sensitive to outliers [27]. RPN loss function for an image was defined as

$$\begin{array}{ccc} L_{RPN} & = & \frac{1}{|S_{R}|}\sum_{i\in S_{R}}L_{CLS}^{R}({\hat{p}}_{i}^{R},p_{i}^{R})+\frac{1}{4\times |S_{R}|}\sum_{i\in S_{R}}p_{i}^{R}\cdot L_{REG}^{R}({\hat{t}}_{i},t_{i}), \end{array}$$ (5)

where

$$\begin{array}{ccc} L_{CLS}^{R}({\hat{p}}_{i}^{R},p_{i}^{R}) & = & -p_{i}^{R}\cdot \text{log}({\hat{p}}_{i}^{R})-(1-p_{i}^{R})\cdot \text{log}(1-{\hat{p}}_{i}^{R}) \end{array}$$ (6)

was the loss function for classification and

$$\begin{array}{ccc} L_{REG}^{R}({\hat{t}}_{i},t_{i}) & = & \sum_{l\in S_{t}}L_{h}({\hat{t}}_{\left(i,l\right)}-t_{\left(i,l\right)}) \end{array}$$ (7)

was the loss function for regression with

$$\begin{array}{ccc} L_{h}\left(t\right) & = & \left\{ \begin{array}{cc} 0.5t^{2} & \text{if}\;|t|<1\\ |t|-0.5 & \text{otherwise}\;. \end{array}\right. \end{array}$$ (8)

being the Huber loss. *i* was the index of anchors sampled during training RPN and *S*_*R*_ was the corresponding collection. ${\hat{p}}_{i}^{R}$ was the predicted probability of anchor *i* being a lesion area. The ground-truth label $p_{i}^{R}$ was 1 if the anchor was a lesion area and was 0 if the anchor was the background. *S*_*t*_ = {*x*, *y*, *w*, *h*}. The classification network consisted of fully connected layers (FCs), which estimated the softmax probabilities for being a lesion area for each RoI. The classification loss $L_{CLS}^{P}$ for each RoI was defined as:

$$\begin{array}{ccc} L_{CLS}^{P}({\hat{p}}_{j}^{P},p_{j}^{P}) & = & -p_{j}^{P}\cdot \text{log}({\hat{p}}_{j}^{P})-\left(1-p_{j}^{P}\right)\cdot \text{log}(1-{\hat{p}}_{j}^{P}), \end{array}$$ (9)

where ${\hat{p}}_{j}^{P}$ was probability for the lesion. The ground-truth label $p_{j}^{P}$ was 1 for RoI being a lesion area and 0 for RoI being the background. *j* was the index of RoIs sampled during training. The mask network was a small FCN applied to each RoI, predicting a segmentation mask in a pixel-to-pixel manner. The mask network output a binary mask for RoI *j* by using a per-pixel sigmoid. The average binary cross-entropy loss $L_{MASK,j}^{p}$ for each RoI was defined as follows:

$$\begin{array}{c} L_{MASK,j}^{P}({\hat{p}}_{k}^{P},p_{k}^{P})=-\frac{p_{j}^{P}}{|S_{j}|}\sum_{k\in S_{j}}(p_{k}^{P}\cdot \text{log}({\hat{p}}_{k}^{P})+(1-p_{k}^{P})\cdot \text{log}(1-{\hat{p}}_{k}^{P})), \end{array}$$ (10)

where ${\hat{p}}_{k}^{P}$ was the predicted probability of pixel *k* in the lesion areas. The ground-truth label $p_{k}^{P}$ was 1 if the pixel was in lesion areas, and was 0 if not. *S*_*j*_ was the collection of pixels in RoI *j*.

The classification loss and the segmentation loss formed the prediction loss. The prediction loss *L*_*PRE*_ was defined as follows:

$$\begin{array}{ccc} L_{PRE} & = & \frac{1}{|S_{P}|}\sum_{j\in S_{P}}(L_{CLS}^{P}({\hat{p}}_{j}^{P},p_{j}^{P})+L_{MASK,j}^{P}({\hat{p}}_{k}^{P},p_{k}^{P})), \end{array}$$ (11)

where *j* was the index of anchors sampled during training prediction. *S*_*P*_ was the corresponding collection. The RPN loss *L*_*RPN*_ and the prediction loss *L*_*PRE*_ can be used jointly or iteratively for training models.

### Training strategy

The PyTorch and torchvision (http://pytorch.org/) were used for model training and evaluation. Our CheXLocNets were trained under the framework of transfer learning [28]. The parameters trained with the COCO dataset [29] were used as the initial parameters of the backbone of CheXLocNets. Consequently, the computational burden caused by training from scratch was able to be saved. Based on this initialization, the training set was further used to fine-tune CheXLocNets. The details about performances of the model training from scratch were shown in S5 and S6 Figs. Before inputting into the network, the radiographs were first converted to RGB images compatible with pre-trained ResNet-50. Then they were normalized based on the mean and standard deviation of images in the ImageNet training set and were flipped horizontally with a 50% probability. Each mini-batch had 4 images and each image had 512 sampled RoIs with a ratio of 1:3 of positives to negatives. We trained six CheXLocNets with various hyperparameters, denoted by CheXLocNet I to VI. Each model training process was divided into three stages with decayed learning rates as 10^−3^, 10^−4^, and 10^−5^. In each stage, the six CheXLocNets were trained with 10 epochs. The network performance on the validation set was evaluated with AP_50_ (average precision at intersection over union (IoU) equal to 0.50) at the end of each epoch. The parameters with the best AP_50_ in the previous stage were used as the initial parameters for the next stage. In the third stage, the optimal parameters were used as the final ones.

CheXLocNet I was trained with an approximate joint training method, whose loss integrated the RPN loss and the mask loss. The optimization was conducted with the Adam algorithm with *β*(0.9, 0.999) and *ε*(10^−8^) [30]. The anchors were set following the work [24]. Then, we changed part of the hyperparameters of CheXLocNet I and achieved the other five models. Unlike Model I (0.5, 1, 2), new aspect ratios of anchors were used for in CheXLocNet II, which were (0.1, 0.2, 0.4, 0.8, 1, 1.25, 2.5, 5, 10). The shapes of lesions were ranging from (0, 10). Most of they focused in (0, 2.5), and seldom in (2.5, 10). The new aspect ratios were more similar to the shapes of the lesions. More details of the lesion shapes are shown in S1 Fig. CheXLocNet III trained with a stochastic gradient descent with momentum (momentum 0.9, without weight decay). We added a weight decay 0.0001 to the Adam optimizer for CheXLocNet IV. In CheXLocNet V, we handled the origin radiographs with gamma correction to make the texture more visible. More detail of the radiograph brightness is shown in S2 Fig. In CheXLocNet VI training epoch, we used an alternating training method. We first trained CheXLocNet with the RPN loss and then with the prediction loss in each epoch.

### Evaluation strategy

**Fig 2:**
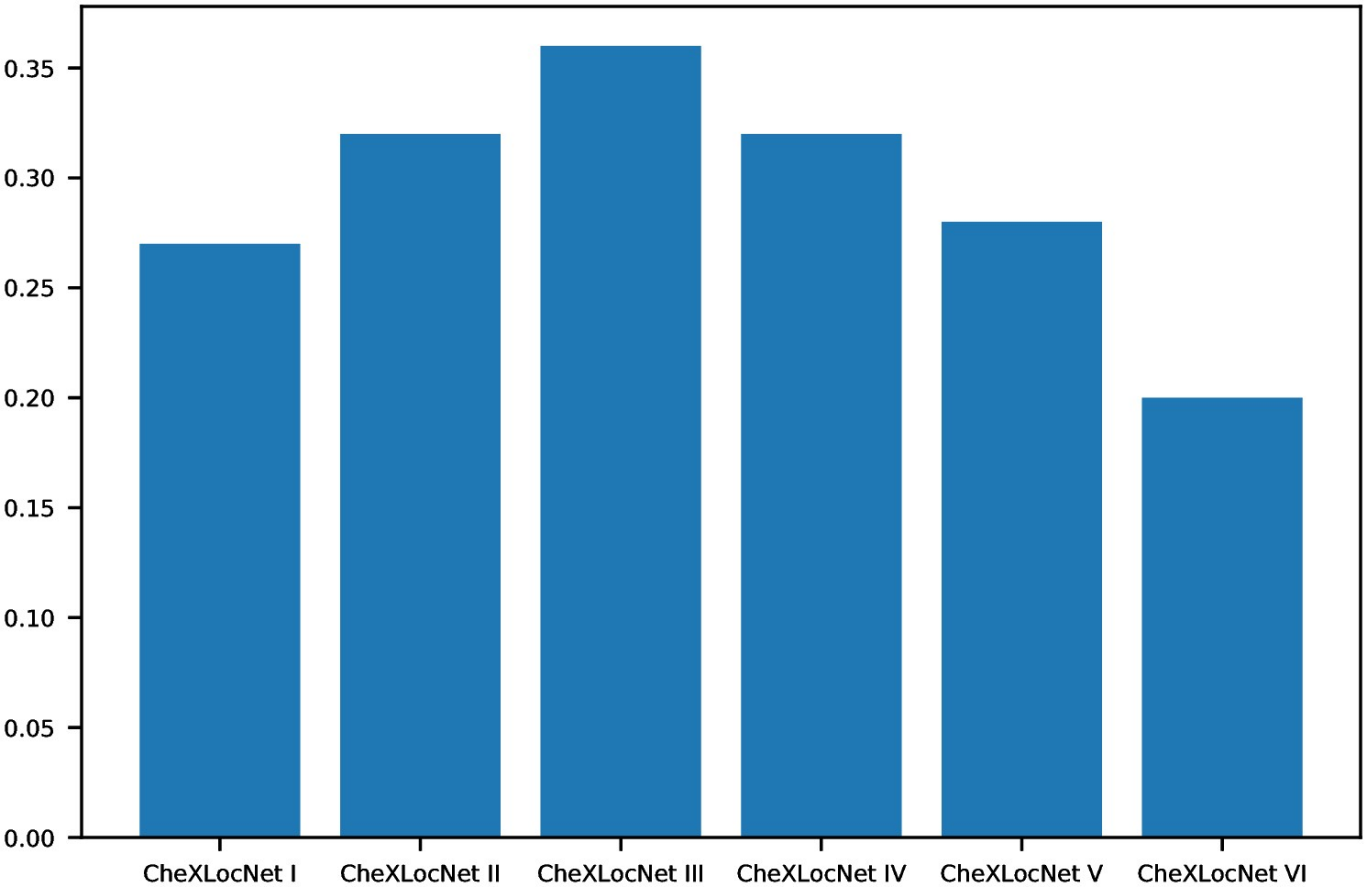
AP_50_ of CheXLocNets. IoU, intersection over union; AP_50_, average precision at IoU = 0.50.

We evaluated the performances of CheXLocNets on the validation set at the end of each epoch. AP_50_ was used to determine the optimal parameters for the lesion segmentation in each stage. We reported the metrics AP_50_ of the performance of the final parameters in Fig 2. AP_50_ measured the precision of the network segmentation performance. The segmentation was considered as a correct segmentation if the IoU of the predicted area and ground truth area exceed 50% [29, 31]. We conducted a comprehensive comparison of classification capabilities of CheXLocNets across 5 performance metrics, including AUC, sensitivity, specificity, F1 score, and positive predictive value (PPV) on the validation set. Statistical comparisons between proportions were performed utilizing z-test and proportion confidence intervals (CIs) were calculated using the Wilson Score confidence interval [32]. All *P* values were assessed with *α* = 0.05. To convert the probabilities produced by CheXLocNets to binary predictions, we chose pathology-specific thresholds through the maximization of the F1 score on the validation set [10]. If the classification probability was less than the thresholds, the predicted mask was treated as empty.

To assess the classification performances of CheXLocNets would generate on the unseen data, we also computed AUC, sensitivity, specificity, and PPV on the testing set. And IoU and Dice score were used to evaluate the segmentation performances of CheXLocNets. IoU, also known as the Jaccard similarity coefficient, is a statistic used for gauging the similarity and diversity of sample sets. IoU can be used to compare the pixel-wise agreement between a predicted segmentation and its corresponding ground truth:

$$\begin{array}{ccc} J(A,B) & = & \frac{\left|A\cap B\right|}{\left|A\cup B\right|}. \end{array}$$ (12)

The Dice coefficient is also a statistic used to gauge the similarity of two samples:

$$\begin{array}{ccc} D(A,B) & = & \frac{2\times |A\cap B|}{\left|A|+|B\right|}. \end{array}$$ (13)

A is the predicted set of pixels and B is the ground truth.

Sensitivity and specificity are statistical measures of the performance of a binary classification test. PPV is the proportions of positive results in statistics and diagnostic tests. The F1 score is used to measure the test accuracy. AUC is equal to the probability that a classifier will rank a randomly chosen positive instance higher than a randomly chosen negative one.

$$\text{sensitivity}=\frac{\text{TP}}{\text{TP}+\text{FN}},$$ (14)

$$\text{specificity}=\frac{\text{TN}}{\text{TN}+\text{FP}},$$ (15)

$$\text{PPV}=\frac{\text{TP}}{\text{TP}+\text{FP}},$$ (16)

$$F1=2\times \frac{\text{PPV}\times \text{sensitivity}}{\text{PPV}+\text{sensitivity}}=\frac{2\text{TP}}{2\text{TP}+\text{FP}+\text{FN}},$$ (17)

where true positive, false positive, true negative, and false negative are denoted as TP, FP, TN, and FN, respectively.

## Results

**Fig 3:**
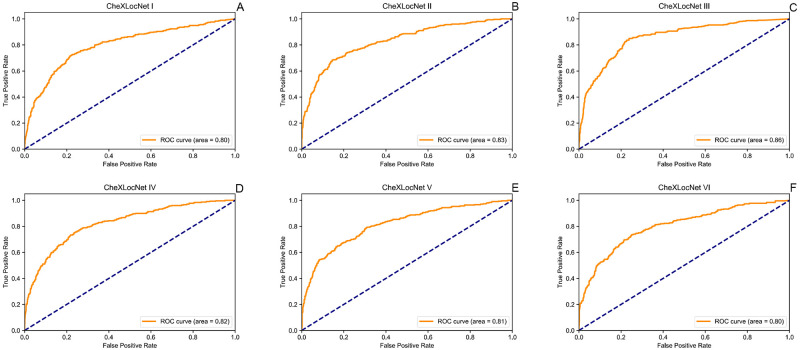
ROC curves of CheXLocNets on validation set. Each plot illustrates the ROC curves of CheXLocNets on the validation set. The ROC curve of the algorithm is generated by varying the discrimination threshold (used to convert the output probabilities to binary predictions). ROC, receiver operating characteristic.

**Fig 4:**
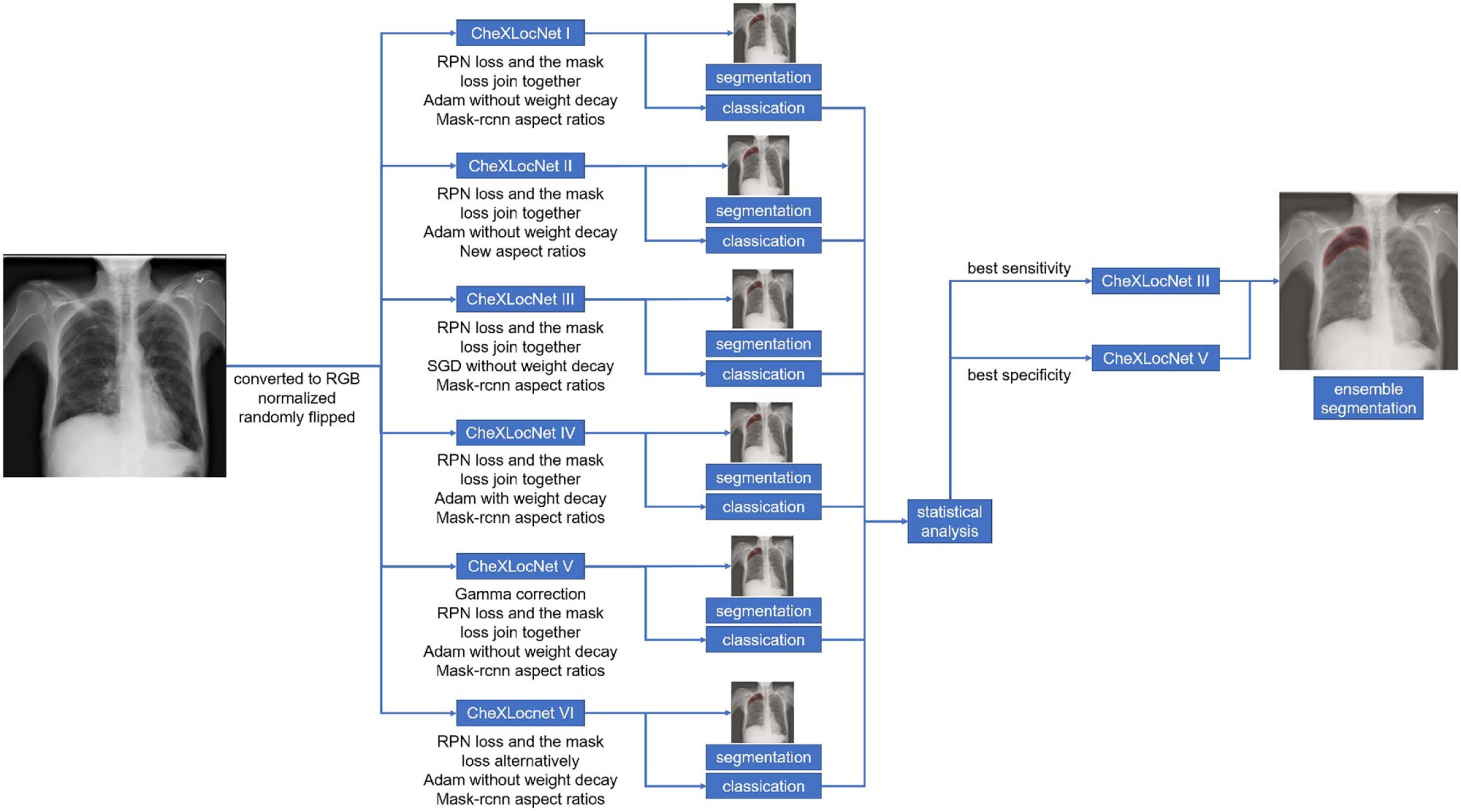
The working procedure of six CheXLocNets. We first trained and evaluated six CheXLocNets separately. Then we selected the two CheXLocNets with the best sensitivity or the best specificity to join together forming an ensemble model.

We trained six CheXLocNets with different procedures separately. The optimal parameters of each network were selected by AP_50_ on the validation set. Those networks had validation AP_50_ ranging from 0.20 to 0.36, illustrated in Fig 2. We used the maximum probability of RoIs being lesions in a radiograph as the classification probability of this radiograph. CheXLocNet III showed the best AP_50_ 0.36. The classification capabilities of CheXLocNets are illustrated in Table 1. The receiver operating characteristic (ROC) curves on the validation set are illustrated in Fig 3. CheXLocNet III achieved the best AUC score 0.86. The F1 scores ranged from 0.57 to 0.64, and CheXLocNet III achieved the best one 0.64. CheXLocNet III also got the best sensitivity performance 0.82 (CI 0.78-0.87). CheXLocNet V showed the best specificity 0.92 (CI 0.90-0.93). Also, we computed the PPV of models ranging from 0.48 to 0.65, and CheXLocNet V achieved the best one 0.65 (CI 0.59-0.71). We selected CheXLocNet III (the CheXLocNet with best sensitivity) and CheXLocNet V (the CheXLocNet with best specificity) to combine an ensemble CheXLocNet to predict the lesion areas. We used a simple approach for ensembling, where we just averaged their masks. The working procedure of six CheXLocNets is shown in Fig 4.

**Table 1:** The classification performance of CheXLocNets on the validation set.

	AUC	F1	sensitivity	specificity	PPV
**CheXLocNet I**	0.80	0.58	0.72 (CI 0.67-0.77)	0.78 (CI 0.76-0.81)	0.49 (CI 0.44-0.53)
**CheXLocNet II**	0.83	0.63	0.68 (CI 0.63-0.74)	0.86 (CI 0.83-0.88)	0.58 (CI 0.53-0.63)
**CheXLocNet III**	**0.86**	**0.64**	**0.82 (CI 0.78-0.87)**	0.78 (CI 0.76-0.81)	0.52 (CI 0.48-0.57)
**CheXLocNet IV**	0.82	0.59	0.66 (CI 0.60-0.71)	0.84 (CI 0.82-0.86)	0.54 (CI 0.49-0.59)
**CheXLocNet V**	0.81	0.59	0.54 (CI 0.49-0.60)	**0.92 (CI 0.90-0.93)**	**0.65 (CI 0.59-0.71)**
**CheXLocNet VI**	0.80	0.57	0.70 (CI 0.64-0.75)	0.79 (CI 0.76-0.81)	0.48 (CI 0.44-0.53)

AUC, area under the receiver operating characteristic curve; CI, confidence interval; PPV, positive predictive value.

**Fig 5:**
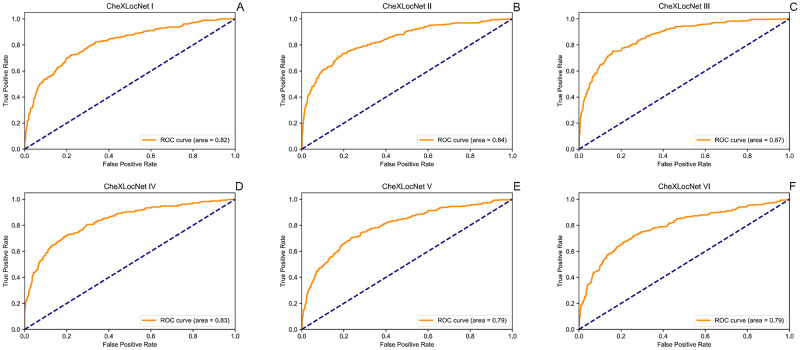
ROC curves of CheXLocNets on testing set. Each plot illustrates the ROC curves of CheXLocNets on the testing set. The ROC curve of the algorithm is generated by varying the discrimination threshold (used to convert the output probabilities to binary predictions). ROC, receiver operating characteristic.

The classification performance was also evaluated on testing set, illustrated in Table 2. The ROC curves on the testing set are illustrated in Fig 5. On the testing set, CheXLocNet III was still the best AUC one. The other networks scores were between 0.79 and 0.84. CheXLocNet III got the best F1 score 0.60. The best sensitivity model was still CheXLocNet III 0.78 (CI 0.73-0.83). The best specificity model was still CheXLocNet V 0.92 (CI 0.90-0.94). The range of PPV scores was [0.47, 0.61], and CheXLocNet V achieved the best one 0.61 (CI 0.54-0.67).

**Table 2:** The classification performance of CheXLocNets on the testing set.

	AUC	F1	sensitivity	specificity	PPV
**CheXLocNet I**	0.82	0.57	0.70 (CI 0.64-0.75)	0.80 (CI 0.78-0.82)	0.48 (CI 0.43-0.53)
**CheXLocNet II**	0.84	0.60	0.63 (CI 0.58-0.69)	0.87 (CI 0.85-0.89)	0.56 (CI 0.51-0.62)
**CheXLocNet III**	**0.87**	**0.60**	**0.78 (CI 0.73-0.83)**	0.78 (CI 0.76-0.81)	0.49 (CI 0.45-0.54)
**CheXLocNet IV**	0.83	0.60	0.66 (CI 0.61-0.72)	0.85 (CI 0.83-0.87)	0.54 (CI 0.49-0.59)
**CheXLocNet V**	0.79	0.53	0.46 (CI 0.40-0.52)	**0.92 (CI 0.90-0.94)**	**0.61 (CI 0.54-0.67)**
**CheXLocNet VI**	0.79	0.55	0.65 (CI 0.60-0.71)	0.80 (CI 0.78-0.83)	0.47 (CI 0.42-0.52)

AUC, area under the receiver operating characteristic curve; CI, confidence interval.

**Fig 6:**
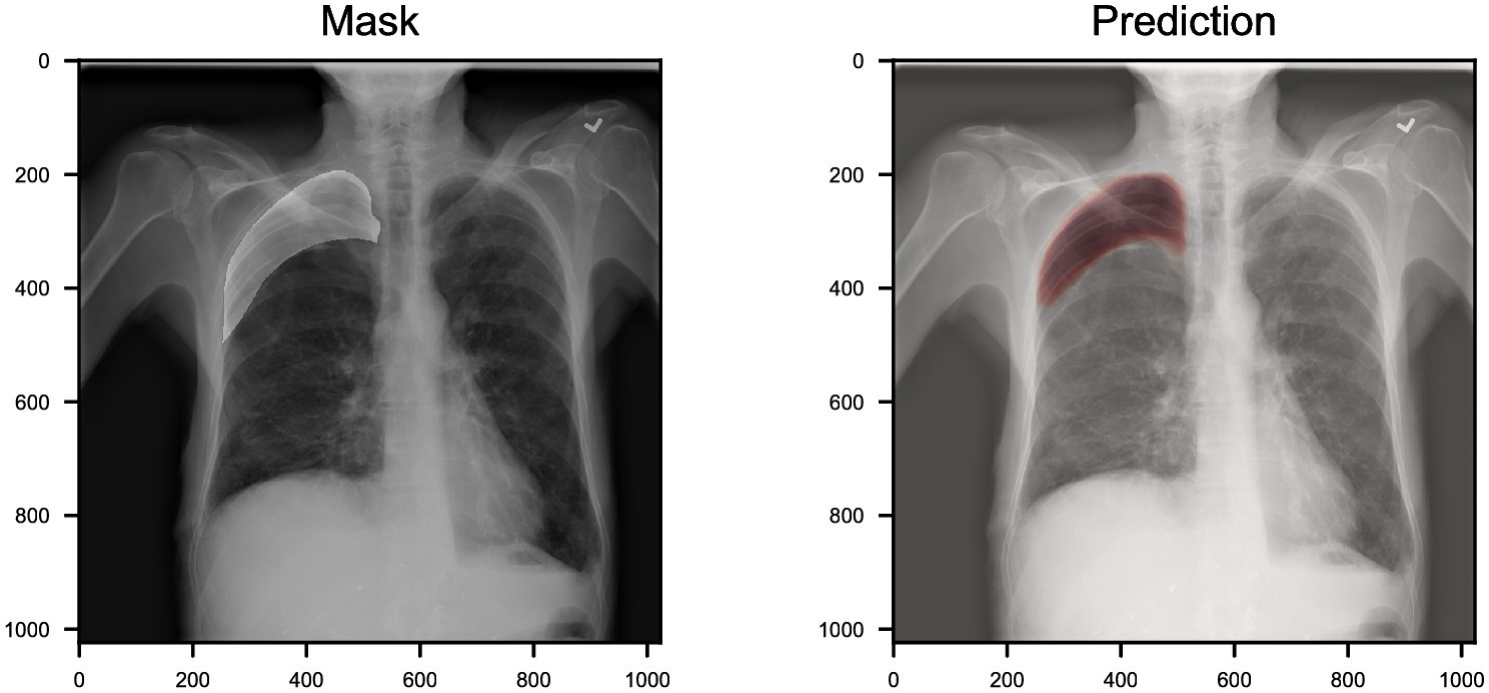
An example of chest radiology report. We highlight the location of the pneumothorax lesion in the chest radiograph (left). The probabilities of segmentation output by CheXLocNet are present in varying shades of red (right). CheXLocNet correctly detected the pneumothorax and masked the lesion area roughly.

To evaluate the performance of CheXLocNets for segmentation, we computed IoU and Dice score of each CheXLocNet on the testing set. CheXLocNet V achieved best segmentation results with IoU 0.77 and Dice score 0.79, among the single CheXLocNets. CheXLocNet II narrowly lost the first place with IoU 0.75 and Dice score 0.77. We also trained other two models, a single U-net with resnet34 as the encoder and AlbuNet [33], for the comparable. AlbuNet is an ensemble model for the single U-net we used. Table 3 shows the comparison between these models. The ensemble CheXLocNet achieved the best result(IoU score 0.81, Dice score 0.82). While above half of our CheXLocNets could not surpass the single U-Net, CheXLocNets V outperformed U-Net 1.1% of IoU score and 1.4% of Dice score. Remarkable improvements have been achieved by ensembling CheXLocNets. Fig 6 shows an example of segmentation prediction. The prediction overlay on the original images is shown on the right. The ground-truth mask overlay on the original images is shown on the left. More examples can be seen in S3 Fig. While achieving good results, CheXLocNet consumed fewer hardware resources and ran faster than the variants of U-net. CheXLocNet consumed 10G GPU memory with batch size 4. The Albunet consumes 10G GPU memory with batch size 2. The elapsed time of training CheXLocNet one epoch was approximate 18 minutes with a Tesla K40m. That of Albunet was approximate 93 minutes with a Tesla K40m.

**Table 3:** The comparison of IoU and Dice score on the testing set.

	Segmentation	
	IoU	Dice
**CheXLocNets I**	0.70	0.72
**CheXLocNets II**	0.75	0.77
**CheXLocNets III**	0.69	0.72
**CheXLocNets IV**	0.74	0.75
**CheXLocNets V**	0.77	0.79
**CheXLocNets VI**	0.69	0.71
**Ensemble CheXLocNet**	**0.81**	**0.82**
**U-Net**	0.76	0.78
**AlbuNet**	0.80	0.80

IoU, (intersection over union).

## Discussion

We proposed CheXLocNet to localize the pneumothorax lesions in the radiographs automatically. Our CheXLocNet produced an IoU of 0.81 and a Dice score of 0.82. Thus, the clinical integration of CheXLocNet can be potentially helpful for the patients who cannot access the medical imaging expertise. After all, it has been reported that more than 4 billion people lack the medical imaging services around the world [34]. This performance makes our CheXLocNet of potential values in two folds. First, CheXLocNet can offer the classification for the radiographs automatically. As indicated by [10], the automatic classification for the radiographs can benefit the worklist prioritization, which permits the most serious patients to receive quicker diagnose. The deep learning aided quick diagnose can also be applied in the emergency department [35]. Furthermore, CheXLocNet offers the segmentation of lesions, which can be used to sequentially monitor the geometric changes of lesions and thereby evaluate the effect of therapy, like adjuvant chemotherapy [36]. Prior studies suggest that perceptual errors and biases can be reduced by providing feedback on the presence and locations of abnormalities on radiographs to interpreting radiologists [37], a scenario that is well suited for our CheXLocNet. Compared with the U-shaped models, CheXLocNet consumed fewer hardware resources and trained faster on the dataset. This makes it more convenient to apply in clinically realistic environments.

Because our CheXLocNet succeeded in the detection of pneumothorax in chest radiographs, it can be expected to effectively extract the chest radiographs texture features. Thus, CheXLocNet can be extended to multi-label segmentation, by replacing the prediction classifier with a multi-classifier. Our CheXLocNet also can be trained for detecting multiple thoracic pathologies, like pneumonia, atelectasis, cardiomegaly, effusion, and etc.

Recently, there have been several works for the radiograph segmentation. The previous researches of accurate radiograph segmentation focused on the segmentation of organs [38, 39]. The segmentations of the lungs were used to aid the diagnosis of COVID-19 [15]. Guendel et al. proposed the location-aware Dense Networks for combining the location of the lesions with the classes of diseases as labels [40]. But the localization were conducted by dividing two lungs into ten parts, which lead to low location resolution. Wang et al., Rajpurkar et al. and Oh et al. obtained the weakly-supervised lesion localization heatmap by extracting weights and feature maps from the classification networks [10, 15, 17]. For the data well labeled, the weakly-supervised methods cannot be compatible with fully-supervised ones [18]. Compared with these work, we developed CheXLocNet with a fully-supervised process. Our CheXLocNet sequentially conducted the classification twice based on the anchor and RoI. The bounding-box regression helped to improve the classification a lot. It eliminated the irrelevant area in the anchor. An example is shown in S4 Fig. As a direct result, the end of CheXLocNet could produce the general segmentation masks for most of the pneumothorax lesion areas with IoU 0.81. Thus, CheXLocNet is competent for the classification and segmentation of radiographs for the well-labeled data.

The limitation of CheXLocNet was the backbone. We used a ResNet-50 as the backbone. Because this ResNet-50 was developed for a large polychromatic image dataset, it could be of superfluous channels and weights for the monochromatic radiographs. Although we used transfer learning and early stopping to limit overfitting, yet the segmentation result of individual CheXLocNet is a little overfitting. By far, we offered the ensemble scheme to prevent this problem. We are working to develop a more suitable structure for our CheXLocNet backbone, as well as the corresponding training procedures.

## Conclusion

We present CheXLocNet, a deep learning algorithm for automatic segmentation of pneumothorax lesion areas in chest radiographs. This technology have the potential to improve healthcare delivery and increase access to chest radiograph expertise for the detection of diseases. Further studies are necessary to determine the feasibility of these outcomes in a prospective clinical setting.

## Supporting information

S1 Fig Details of the lesion shapes. The distribution of the width and the aspect ratio of lesions are shown in A and B. Their relationship is shown in C.
(TIF)

S2 Fig Detail information on radiograph brightness. The further visible texture of the radiograph can help the radiologist make a better diagnosis. We applied gamma correction to the radiographs.
(TIF)

S3 Fig Interpreting network predictions. We highlight the location of the pneumothorax lesion in the chest radiograph (left). The probabilities of segmentation output by CheXLocNet are present by red (right).
(TIF)

S4 Fig A sample of RPN bounding-box regression. The bounding-box regression helped to improve the classification a lot. It eliminated irrelevant areas in the anchor. The red rectangle is the true target box. The green one is the anchor box. The blue one is the corresponding RoI. After the bounding-box regression, the rectangle region contained less area than lesions. The possibility of this rectangle declined from 0.94 to 0.27.
(TIF)

S5 Fig The performance of CheXLocNet training from scratch on the validation set. We trained a new CheXLocNet, named CheXLocNet S, from scratch with 1000 epochs. CheXLocNet S was with the same struct as the CheXLocNet I. The initial learning rate was 0.001 and multiplied by 0.1 after 100 epochs. AUC, area under the receiver operating characteristic curve; PPV, positive predictive value; IoU, intersection over union; AP50, average precision at IoU = 0.50.
(TIF)

S6 Fig The performance of CheXLocNet training from scratch on the testing set. We trained a new CheXLocNet, named CheXLocNet S, from scratch with 1000 epochs. CheXLocNet S was with the same struct as the CheXLocNet I. The initial learning rate was 0.001 and multiplied by 0.1 after 100 epochs. AUC, area under the receiver operating characteristic curve; PPV, positive predictive value; IoU, intersection over union; AP50, average precision at IoU = 0.50.
(TIF)
