# Supplementary figures and images for: CheXLocNet: Automatic localization of pneumothorax in chest radiographs using deep convolutional neural networks
Source: PLoS One. 2020 Nov 9;15(11):e0242013. doi: 10.1371/journal.pone.0242013 (PMC7652331; doi:10.1371/journal.pone.0242013)

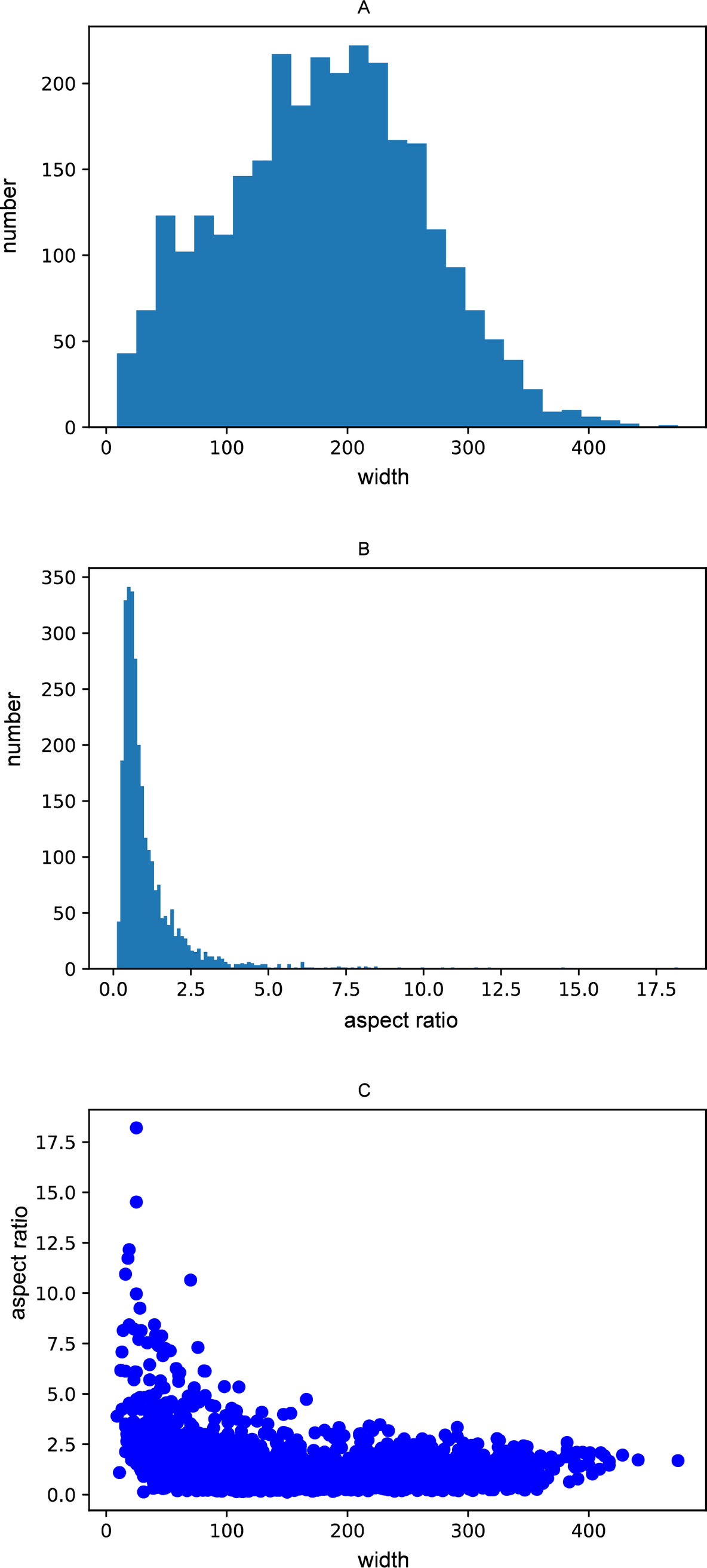

Supplement: S1 Fig — The distribution of the width and the aspect ratio of lesions are shown in A and B. Their relationship is shown in C. (TIF) [file pone.0242013.s001.tif]

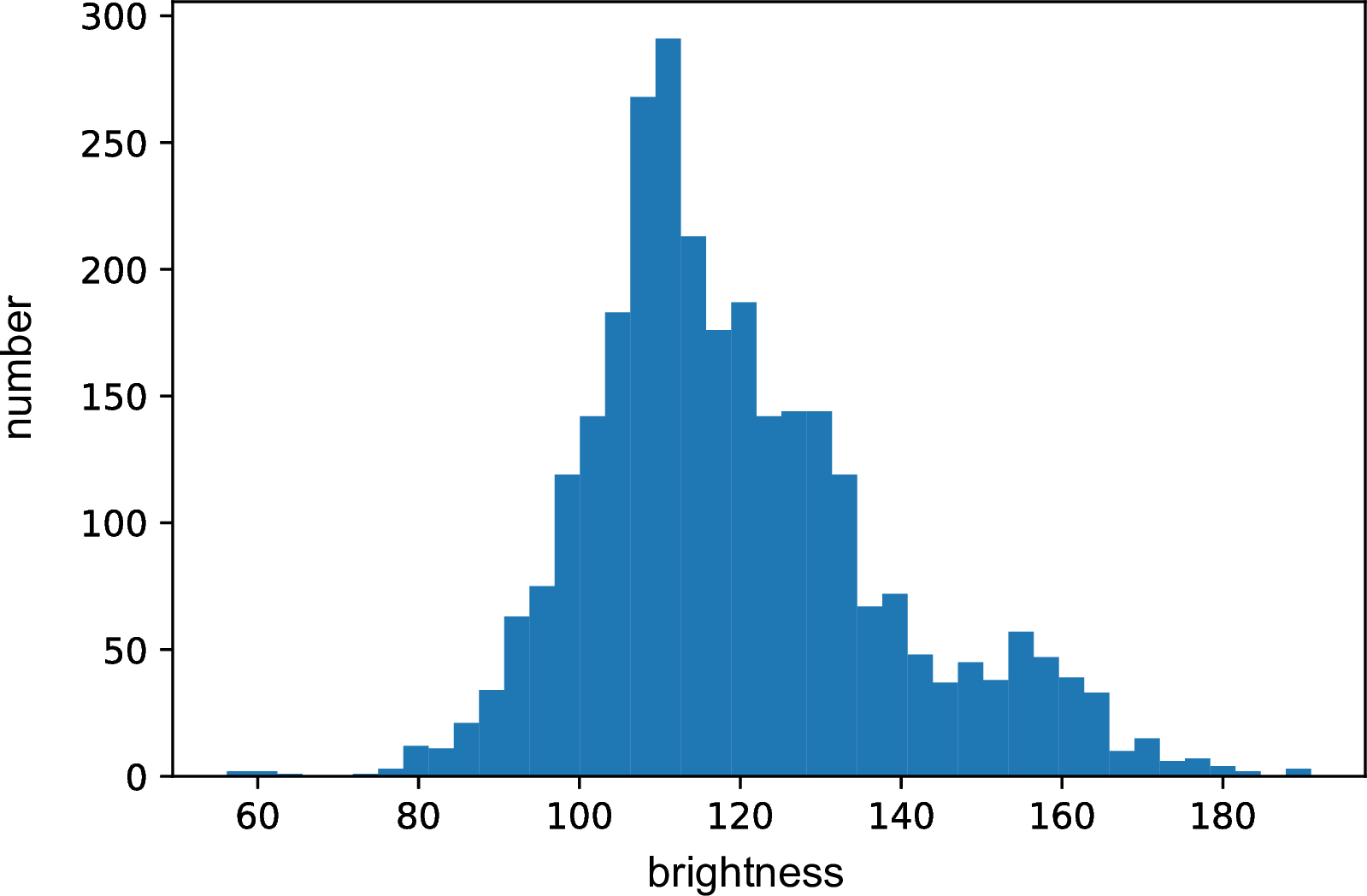

Supplement: S2 Fig — The further visible texture of the radiograph can help the radiologist make a better diagnosis. We applied gamma correction to the radiographs. (TIF) [file pone.0242013.s002.tif]

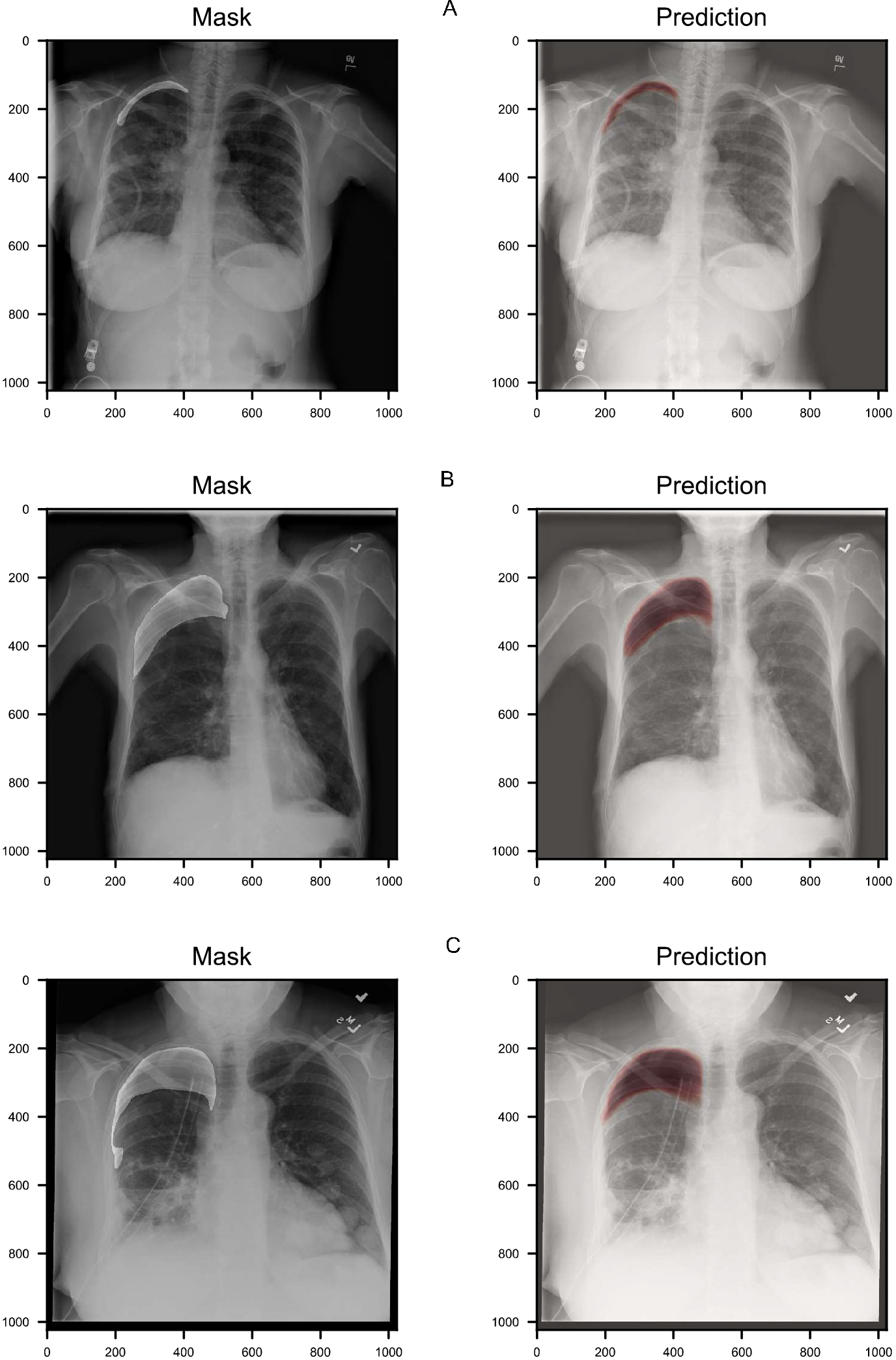

Supplement: S3 Fig — We highlight the location of the pneumothorax lesion in the chest radiograph (left). The probabilities of segmentation output by CheXLocNet are present by red (right). (TIF) [file pone.0242013.s003.tif]

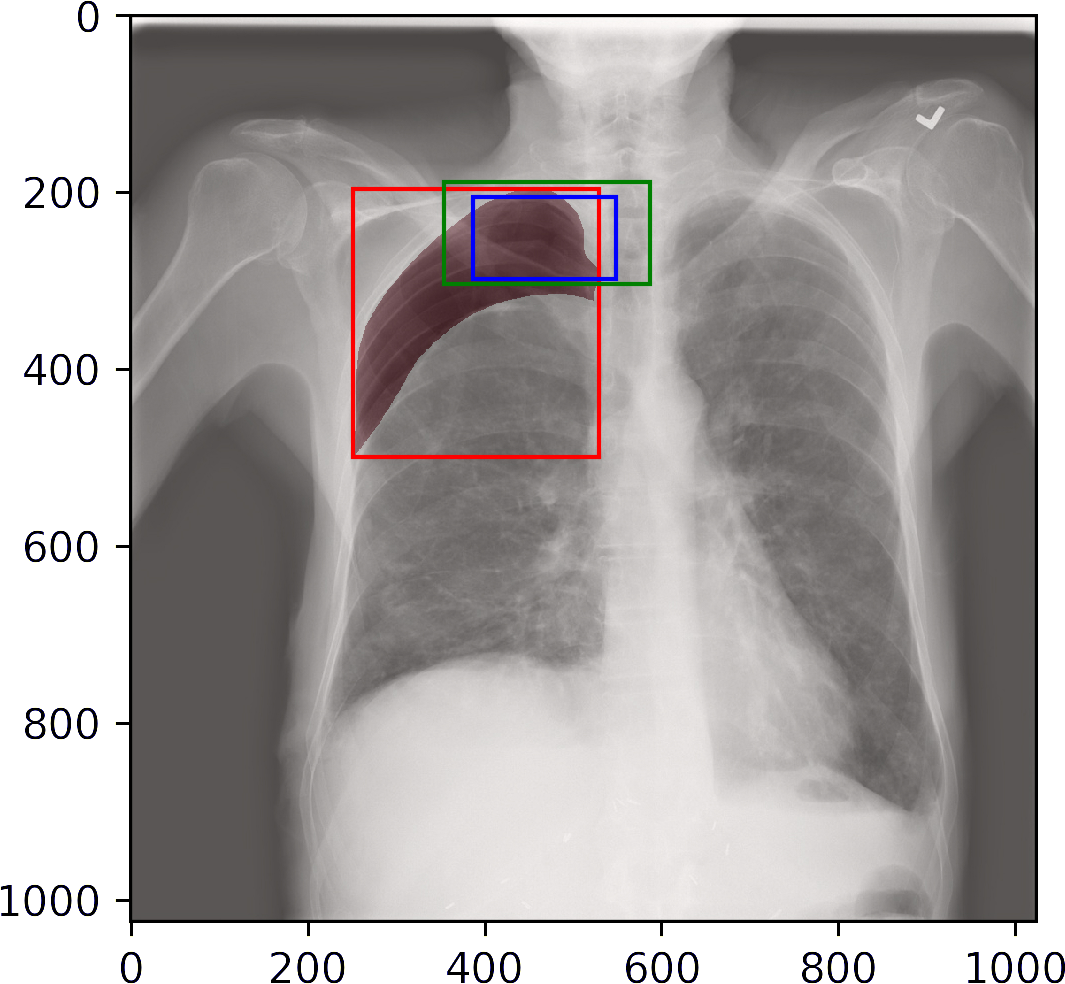

Supplement: S4 Fig — The bounding-box regression helped to improve the classification a lot. It eliminated irrelevant areas in the anchor. The red rectangle is the true target box. The green one is the anchor box. The blue one is the corresponding RoI. After the bounding-box regression, the rectangle region contained less area than lesions. The possibility of this rectangle declined from 0.94 to 0.27. (TIF) [file pone.0242013.s004.tif]

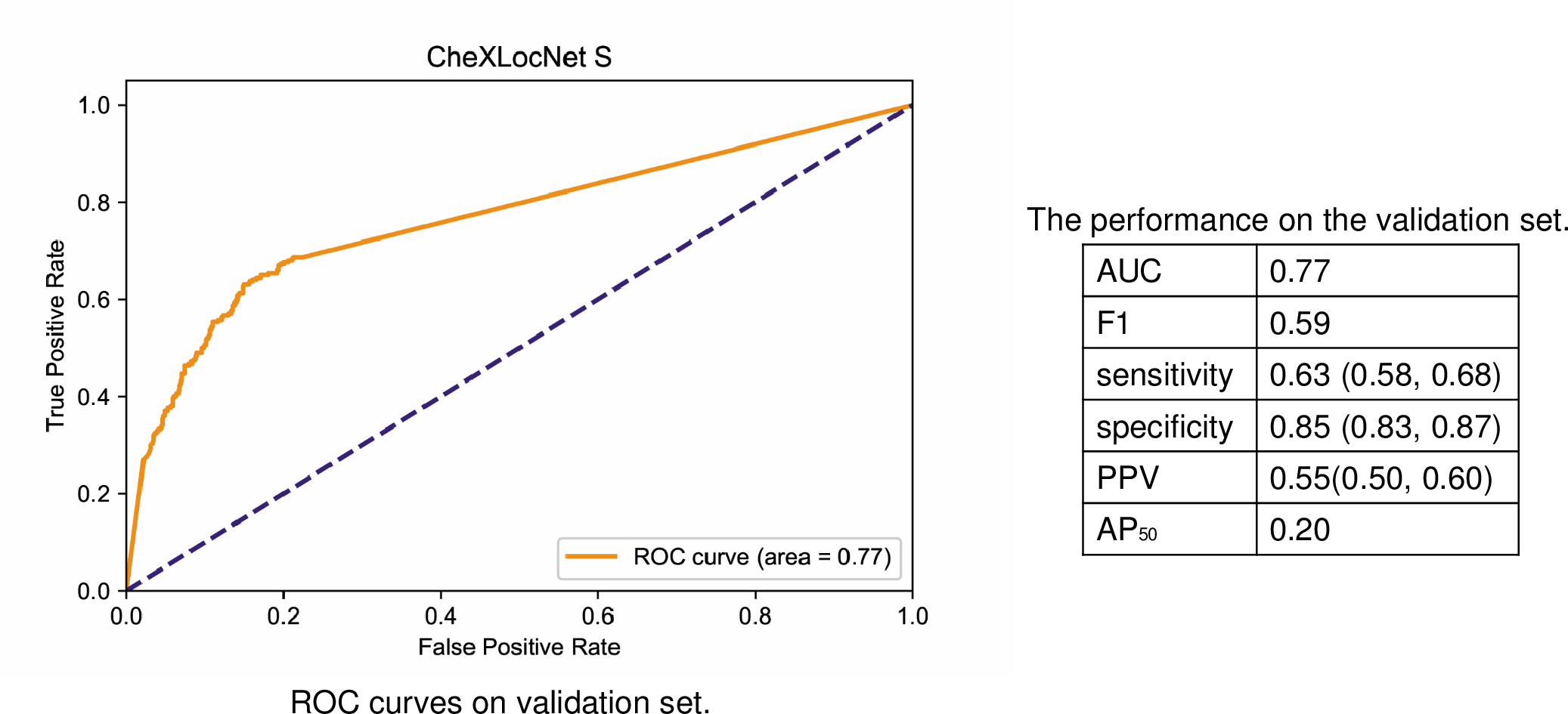

Supplement: S5 Fig — We trained a new CheXLocNet, named CheXLocNet S, from scratch with 1000 epochs. CheXLocNet S was with the same struct as the CheXLocNet I. The initial learning rate was 0.001 and multiplied by 0.1 after 100 epochs. AUC, area under the receiver operating characteristic curve; PPV, positive predictive value; IoU, intersection over union; AP50, average precision at IoU = 0.50. (TIF) [file pone.0242013.s005.tif]

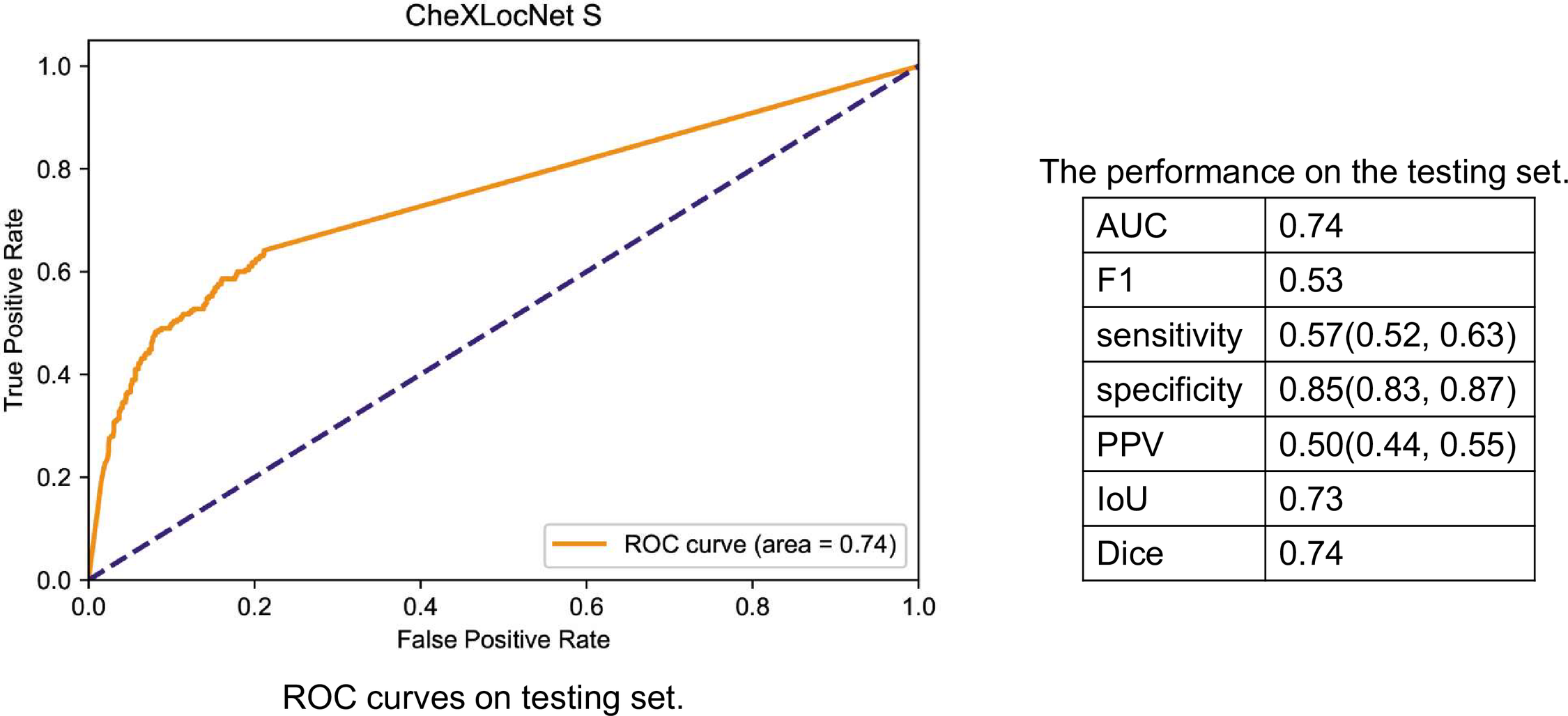

Supplement: S6 Fig — We trained a new CheXLocNet, named CheXLocNet S, from scratch with 1000 epochs. CheXLocNet S was with the same struct as the CheXLocNet I. The initial learning rate was 0.001 and multiplied by 0.1 after 100 epochs. AUC, area under the receiver operating characteristic curve; PPV, positive predictive value; IoU, intersection over union; AP50, average precision at IoU = 0.50. (TIF) [file pone.0242013.s006.tif]
